# How Can We Compare Cochlear Implant Systems across Manufacturers? A Scoping Review of Recent Literature

**DOI:** 10.3390/audiolres13050067

**Published:** 2023-10-17

**Authors:** Elinor Tzvi-Minker, Andreas Keck

**Affiliations:** Syte Institute, Hohe Bleichen 8, 20354 Hamburg, Germany; andreas.keck@syte-institute.com

**Keywords:** cochlear implant, patient-reported outcomes, pure-tone average, speech in noise, music perception

## Abstract

Electric stimulation via a cochlear implant (CI) enables people with severe-to-profound sensorineural hearing loss to regain speech understanding and music appreciation and, thus, allow them to actively engage in social life. Three main manufacturers (Cochlear^TM^, MED-EL^TM^, and Advanced Bionics^TM^ “AB”) have been offering CI systems, thus challenging CI recipients and otolaryngologists with a difficult decision as currently no comprehensive overview or meta-analysis on performance outcomes following CI implantation is available. The main goals of this scoping review were to (1) map the literature on speech and music performance outcomes and to (2) find whether studies have performed outcome comparisons between devices of different manufacturers. To this end, a literature search was conducted to find studies that address speech and music outcomes in CI recipients. From a total of 1592 papers, 188 paper abstracts were analyzed and 147 articles were found suitable for an examination of full text. From these, 42 studies were included for synthesis. A total of 16 studies used the consonant-nucleus-consonant (CNC) word recognition test in quiet at 60 db SPL. We found that aside from technical comparisons, very few publications compared speech outcomes across manufacturers of CI systems. However, evidence suggests that these data are available in large CI centers in Germany and the US. Future studies should therefore leverage large data cohorts to perform such comparisons, which could provide critical evaluation criteria and assist both CI recipients and otolaryngologists to make informed performance-based decisions.

## 1. Introduction

Cochlear implants (CI) encompass implanted electronics and external sound processors that can deliver electric stimulation to the auditory nerve and improve hearing in subjects with severe-to-profound hearing loss. Despite being a well-established intervention for this condition, there is a strong variability in individual hearing restoration achieved by CI, which may depend on several factors ranging from device specifications to surgical placement of the implant, as well as patient-specific factors such as age at implantation and duration of hearing loss [1]. Implant recipients improve their conversational speech understanding following implantation, on average by up to 52% [2], and in many cases, their hearing improves so significantly that they can understand conversational speech in difficult listening situations [3]. Significant improvement in music perception and satisfaction following CI implantation is also observed [4]. This has become particularly important as studies have shown that quality of musical sound and patient QoL post-implantation are significantly associated. However, CI were initially designed to enhance speech discrimination. In the past 10 years, fine structure information has been represented in CI processing strategies to further improve music perception [5].

Recent estimations suggest that approx. one million cochlear implants have been implanted worldwide [6]. Clearly, a rapid growth in this field can be observed as previous FDA (United States Food and Drug Administration) reports from 2019 and 2016 suggest an approx. of 736 K and 324 K resp. implanted devices worldwide. A portion of this growth can be attributed to the expansion of CI candidacy guidelines [7]. Implantation is now available to a broader group of individuals compared to when implants were first introduced in the 1970s. Individuals are now more commonly implanted with a CI system when they suffer from unilateral deafness [8] or intractable tinnitus [9], and in the presence of increasing amounts of residual hearing [7].

Accordingly, the number of scientific studies in the field of CI has increased exponentially as implants have become widely available and candidacy guidelines have expanded. A recent search for cochlear implants on PubMed yielded over 1500 papers in 2021 alone, an increase of almost 600% in the last 20 years (2001: 262 papers). Study topics in the peer-reviewed literature range widely, from candidacy guidelines to implant technological features, programming (mapping), rehabilitation, and performance outcomes, to name a few.

Since the 1990s, four main manufacturers, Cochlear (NSW, Australia), Advanced Bionics (“AB”, Staefa, Switzerland), Oticon Medical (previously Neurelec, Vallauris Cedex, France, and recently acquired by Cochlear), and MED-EL (Innsbruck, Austria), have been offering CI systems. CI users can select from a range of commercially available technology and, therefore, require an objective performance comparison between the different CI systems. A group of CI users who are implanted with CI systems of different brands in each ear are able to compare sound quality and performance; however, these subjects are rare. A study by Harris et al. [10] evaluated speech and music perception using two different brands of CI in the same subjects. Six subjects were implanted with a Cochlear Nucleus in one ear and subsequently implanted with a MED-EL Sonata in the contralateral ear. While no difference in perception was seen in objective testing, subjective music perception was found to be superior with the MED-EL implant in most subjects. Thus, performance comparisons between brands are important and have a strong effect on the quality of life of CI users.

Clearly the differences between CI systems include specific technological features such an electrode array design [11] and speech coding strategies, as well as differences in overall reliability [12], which are of high importance. However, in this scoping review, we aim to highlight the difference in performance outcomes following CI implantation of systems by different manufacturers. This is an aspect that is usually overlooked in comparative studies and should gain more attention. Indeed, in the last decade, patient-reported outcomes (PROs) have gained importance in guiding advancements in medical technology and influenced healthcare policy and practice [13]. A recent review [14] focusing on PROs of implanted devices found 47 studies in the last 22 years that report PROs for CI, including speech and music perception outcomes. However, only 17 studies out of these 47 provided any information on device manufacturers. This means that the data needed to compare speech and music performance outcomes between different devices apparently exist. Whether comparisons have been made is what we aim to uncover in this review.

One outstanding example, dating back to 15 years ago, is a study by [15], which investigated patients’ performance in monosyllabic word tests presented in quiet and under different noise levels, and compared CI systems by three manufacturers (Cochlear, AB and MED-EL). The results showed differences between devices in vowel recognition and sentence comprehension in noise. In addition, the authors showed that when the input dynamic range was increased, the performance measures of all devices improved. A later study by Haumann et al. [16] compared speech performance in noise under realistic adaptive conditions across five different CI systems (Freedom and Esprit 3G by Cochlear, Auria and Harmony by AB, Opus 2 by MED-EL). Here, the results showed a clear disadvantage for Freedom (Cochlear) compared to Opus 2 (MED-EL). Other studies have compared specific technical features of these systems [17,18]. For example, Killan et al. [19] assessed the effect of inter-implant interval and onset of profound deafness based on sound-source localization in children with bilateral cochlear implants from three manufacturers (Cochlear, AB and MED-EL). The authors found that MED-EL devices were associated with significantly better sound-source localization when compared to both Cochlear and AB devices.

A recent retrospective study by Sturm et al. [20] investigated the effect of physical features of CI electrodes, including length and shape of the electrode array, as well as its position relative to the cochlear modiolus, on hearing outcomes. The authors recruited 119 adult (>18 years) subjects with post-lingual hearing loss, who underwent cochlear implantation with a full electrode array insertion. Seven different electrode arrays from three CI manufacturers (Cochlear, MED-EL, AB) were compared. Speech perception outcomes were measured using the consonant-nucleus-consonant (CNC) word recognition test in quiet at the same presentation level and fixed test intervals (3, 6, 12, and 24 months following implantation). Pre-operative speech scores were similar between electrode array groups and the same surgical approach was used. Given the consistencies in data collection and patient demographics, this study was well configured for a comparison between devices. However, the authors chose to compare speech performance without accounting for pre-operative measures of CNC and, thus, dramatically increased inter-individual variability as well as reduced the reliability of device comparisons. In addition to pre-operative speech perception score, several other factors should have been accounted for, such as (1) the duration of deafness and pre-operative hearing aid used, and (2) the cochlear duct length, insertion angle, and electrode position within the cochlea. Lastly, while CNC in quiet may provide some insights on hearing abilities, other speech tests performed in noise, thus assimilating real-life scenarios, are much more suitable for assessing the range of auditory abilities following implantation.

A review by Boisvert et al. [21] further supports the possibility to conduct comparative studies across CI manufacturers. The authors aimed to provide evidence for the efficacy of unilateral cochlear implantation in adults by assessing the procedure’s success rate based on speech perception or self-reported measures in studies from the last 22 years. The authors found that measurements, research design, and reporting of results were inconsistent, with 46 studies using monosyllabic words for post-operative speech perception tests, while 34 studies used sentences in quiet to test for speech perception following CI implantation. In addition, there was some relative consistency with the presentation levels of monosyllabic words in quiet, with most studies using 60 dB SPL (32% of studies) or 65 dB SPL (36% of studies). Such numbers suggest potential for a meaningful comparison of speech outcomes between devices of different manufacturers.

To be able to compare data from existing publications and determine if speech and music performance outcomes using a specific CI system is superior, several factors need to be considered. Firstly, it would be important to compare devices of the same generation. As CI technology advances, patient performance improves [22]. Therefore, a comparison of outcomes with the latest technology to previous-generation devices is not sensible. Another consideration is the test conditions. Ideally, identical conditions are necessary to systematically compare performance outcomes. Evaluation tools, presentation levels, signal-to-noise ratios, language, and test intervals would need to be similar, if not identical, to be able to perform meaningful comparisons [23]. Lastly, subject demographics must be considered. Duration of deafness, prior use of amplification, pre-operative pure-tone thresholds (PTA), and certain etiologies are all known factors that may impact speech performance following implantation [24]. Finally, similar patient profiles would be important in isolating the effect of CI systems that drives outcome differences and, therefore, allow an optimal comparison between CI systems of different manufacturers.

The main goals of this scoping review were to (1) map the literature on speech and music performance outcomes and to (2) find whether studies have performed outcome comparisons between devices of different manufacturers. Manufacturer comparisons focusing on outcomes are of crucial importance for clinicians, CI candidates, and manufacturers. Such comparisons could be an important, more straightforward, and reliable source for decision-making processes when comparing various technical device features that differ between CI systems (https://cochlearimplanthelp.com/cochlear-implant-comparison-chart/, accessed on 16 October 2023). CI manufacturers could benefit from this transparency by better understanding the effect of technological advancements on patient outcomes and factoring these key learnings into future developments. Evidence suggests that more competitive markets within the healthcare industry lead to increased quality of product features [25].

## 2. Methods

A scoping review methodology was chosen to map the literature on speech and music perception outcomes in adult CI users and to find whether studies have compared performance outcomes across CI manufacturers. A scoping review is ideal for answering these types of questions as it provides coverage of a body of literature on a given topic, thus giving a clear indication of the availability of studies [26]. We applied the Population, Concept, and Context (PCC) framework recommended for scoping reviews, which guided the protocol listed below. The population is hearing-impaired adults who underwent implantation of a CI system. The concept of the scoping review is speech and music perception outcomes, and the context is defined to be the availability of CI manufacturer information.

### 2.1. Search Strategies

**Figure 1:**
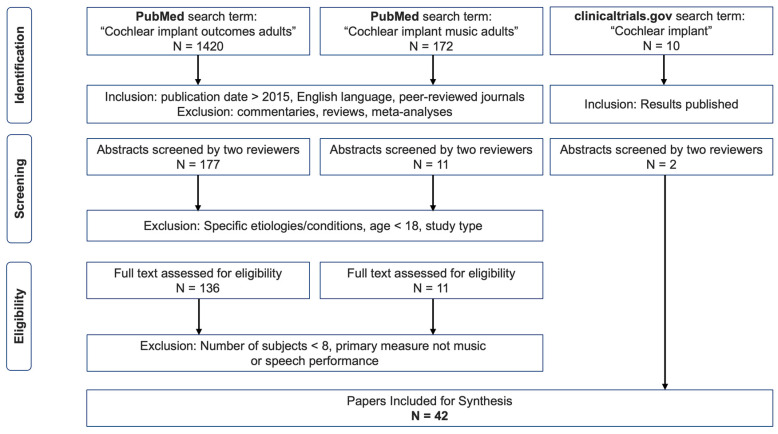
Literature inclusion flowchart.

A literature search was conducted using both PubMed database and Google Scholar search engine, thus covering a broad literature source for the field of cochlear implants. We used the key words “Cochlear implant outcomes adults” (S1) as well as “Cochlear implant music adults” (S2) in March 2022. The review protocol was not pre-registered. An initial search found that Google Scholar showed less relevant publication titles when compared to PubMed, and the publications found in the former matched those found in PubMed. In addition, we searched the clinicaltrials.gov database, which provides information on funded clinical trials around the world, using the search term “Cochlear Implant” (S3) in March 2022. We cross-checked the findings with the first 200 papers on Google Scholar. Figure 1 describes our screening procedure.

### 2.2. Eligibility Criteria

We independently selected all English-language, peer-reviewed studies published after 2015. This time frame was selected to include speech and/or music perception outcomes of the latest technology as comparisons to previous-generation devices is not sensible (see the Introduction section for further explanation). This resulted in 1420 papers for S1 and 172 papers for S2. Then, we discarded all reviews, commentary articles, case-studies, and meta-analyses as these do not contain detailed information required for our scoping review (mainly speech and music perception scores and manufacturer information). Next, the abstracts of all remaining studies (S1 = 177, S2 = 11) were analyzed independently and charted using Excel (Version: 16.77.1, © Microsoft Office, Redmond, WA, USA). The authors applied the following exclusion criteria to the remaining studies through a mutual discussion.

- Exclusion based on indication:

Reports on populations with the same etiologies/conditions, e.g., Meniere’s disease, auditory deprivation, vestibular schwannomas, active military duty, prelingually deaf, or cognitive decline, were excluded as these factors are known to impact performance.

- Exclusion based on participant-specific criteria such as age < 18
- Exclusion based on study type:

Studies focusing on predicting factors that could influence performance outcome, such as genetics, fatigue, subject self-reports, candidacy, robotics, telemedicine, auditory training, surgical approaches, reimplantation, surgical complications, or revision surgery, were excluded to eliminate the inclusion of very specific populations that may differentially affect performance and are not representative of the CI community. Longitudinal studies were excluded as they include previous-generation devices. Comparison of CI performance with other technologies (hearing aids, bone conduction implants) and studies on drug therapies provided in addition to implantation were excluded. Studies that used objective measures not accompanied by speech scores or measured listening effort or hearing preservation as a primary outcome measure were also excluded.

Following this exclusion, a total of *n* = 147 (S1 = 136, S2 = 11) publications were found to be suitable for further analysis and an examination of the full text. We then additionally excluded studies with number of subjects lower than eight and primary measures that were not speech or music performance. Following this in-depth review of studies, we found 42 publications that we included in the final overview table (see Supplementary Materials). Note that until this stage, the availability of manufacturer information had not been assessed.

### 2.3. Data Charting Process

A data-charting form was created in Excel by two reviewers (AK, ETM). Variables to be extracted from the studies were determined by all authors. Data were extracted as reported in the text or figures. Study authors were not contacted when study information was unclear or not reported. The data charting categories included general information such as the publication title, authors’ names, institution where data were collected, and year and journal of publication. In addition, device-specific information was collected such as device type and launch date, as well as manufacturer information. Note that no studies with bi-branded CI recipients were included. Study characteristics such as the number of participants included in the study, age range, and gender, as well as pre-operative parameters, such as pure-tone average (PTA) and duration of hearing loss, were recorded. Importantly, we charted detailed information on the specific post-operative speech or music performance test used, including test conditions, such as whether it was performed in noise and the test interval following CI implantation.

### 2.4. Synthesis of Results

We mapped the findings based on the following criteria: (1) study characteristics, including publication year and journal, (2) manufacturer information and launch data (3) speech tests, (4) speech test conditions, (5) music performance, and (6) number of subjects (histogram). Analysis of speech perception outcomes focused on the most used test: CNC word recognition. The data from six studies that used CNC words presented at 60 dB SPL in quiet were further compared. Three of these studies reported using devices manufactured by Cochlear, and three reported using devices manufactured by MED-EL. To account for the different number of participants in each study included in this analysis, we performed weighted averaging of the scores by the number of participants. We synthesized the results for different intervals including at 1 month, 3 months, 6 months, and 12 months following implant operation.

## 3. Results

### 3.1. Study Characteristics

The 42 studies included are detailed in the Supplementary Materials. Most studies were published in 2020 (*n* = 9) and a relatively low number of studies were published in 2017 (*n* = 3) and 2021 (*n* = 4). Most studies were published in *Otology & Neurotology* (*n* = 9), followed by *Cochlear Implants International* (*n* = 6). Only three studies were published in *JAMA Otolaryngol Head Neck Surg*.

### 3.2. CI System Manufacturer Information

**Figure 2:**
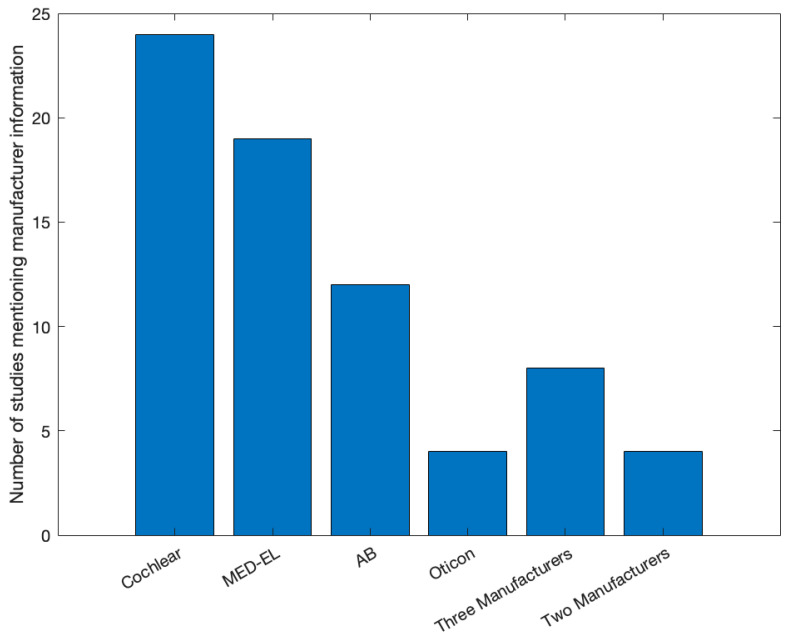
The bar plot shows the number of studies identified for each CI manufacturer and when 2 or 3 manufacturers were mentioned.

Only two studies out of the 42 studies did not mention any information regarding the manufacturer of the CI systems used in the study. For the remaining 40 studies, 5 studies did not mention any information on the specific model of the CI system. The distribution of studies mentioning specific CI systems is shown in Figure 2. Most studies mentioned Cochlear as a manufacturer for the CI systems implanted in the included cohort (*n* = 24). MED-EL was mentioned by 19 studies, AB by 12, and Oticon by 3. Five studies mentioned three manufacturers, and five studies mentioned two manufacturers.

### 3.3. CI System Launch Date

Of the studies mentioning a manufacturer, we noted the device types to compare the date of study publication with the novelty of the device technology. Only 24 studies (out of 42, 57%) mentioned the device type. On average, CI systems were seven years old when these studies were published. For studies including different manufacturers, the differences in launch dates were as much as 23 years (e.g., [27]). Thus, direct comparisons in such studies were deemed invalid.

### 3.4. Sample Size and Post Hoc Power Analysis

The total number of subjects ranged from 8 to 2247, with two studies having less than 10 subjects investigated. Most studies had between 10 and 20 subjects investigated. Only 2 out of the 42 studies that we investigated provided a power analysis to estimate the sample size needed. Neben et al. [28] evaluated the number of subjects needed for speech perception performance test in noise based on a previous hybrid hearing study [29], which determined a 1.1 dB difference to be clinically relevant. They found that 20 subjects were needed to reach a power of 83% for within-subject comparisons. The other study by [30] estimated an effect size of 0.56 for bimodal CI users, which would require 21 subjects for within-group comparisons at 80% power. However, to assess speech performance differences between CI systems of different manufacturers, a between-group comparison is required. Here, we estimated how many patients would be needed to perform valid comparisons between CI manufacturers based on the results of a previous statistical power analysis [20]. These estimates could guide future studies that wish to perform comparisons within one or two CI centers but are concerned with the statistical power of such comparisons. Significant differences in CNC score were found at 3 and 6 months post-implantation using standard *t*-tests. Given the data (means and standard deviations) provided in the paper, and assuming a power of 95% and alpha of *p* = 0.05, we found an effect size of 1.08 for 3-month comparison and 1.04 for 6-month comparison. Based on these rather large effect sizes, we estimated that a minimum total sample size of 42 subjects would be needed to find significant effects when using a parametric Wilcoxon–Mann–Whitney test for comparing differences between two groups (i.e., two manufacturers). The analysis was performed using G*Power [31]. Of the studies that included more than two manufacturers (*n* = 8, see above), only two studies had more than 42 subjects included, one of which was the one this power analysis was based on. The other study by Bruns et al. [32] could have potentially performed a manufacturer comparison with enough statistical power.

#### 3.4.1. Pre-Operative Characteristics

**Age.** The mean or median age at implantation was reported in 83% of the studies (35 out of 42). Two studies reported only the age range. The mean age across the articles was 56.8 ± 9.9. The median was 59 (min: 25, max: 74).

**Figure 3:**
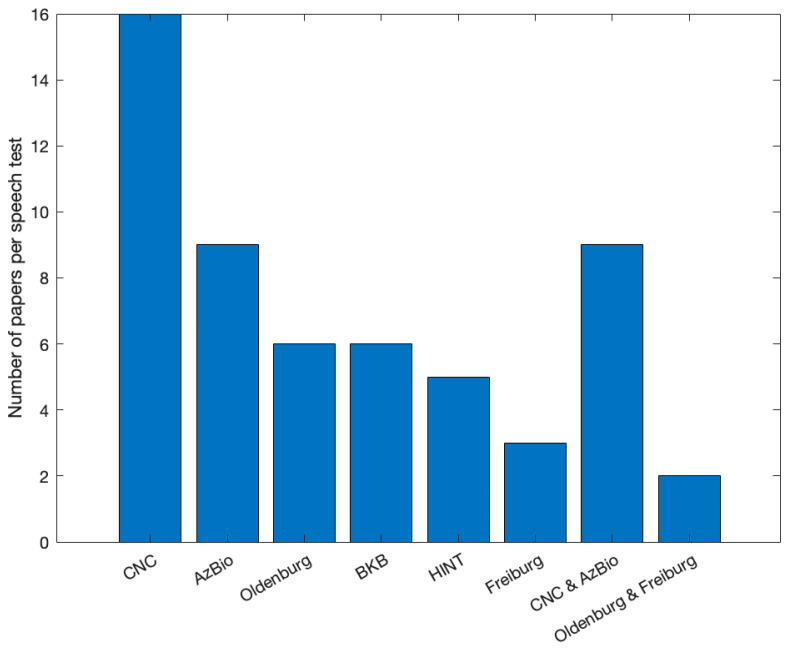
Distribution of speech tests with respect to the number of reviewed studies.

**Etiology.** The articles included in this review only reported on adults with postlingual hearing loss as per the exclusion criteria. Only 50% of the studies reported the etiology of hearing loss in the investigated cohort. Notably, in all studies, unknown etiology was also reported, which accounted for an average of 46.9% of the patients. In Figure 3, we show the number of studies per each etiology in the other 50%. Most studies mentioned a genetic/familial cause (*n* = 13). Next, meningitis, head trauma, ototoxicity, and Meniere’s disease were mentioned in 6–8 studies. Measles appeared in two studies only.

**Duration of hearing loss.** Only postlingual hearing loss was included in this scoping review. Results were obtained for each affected ear and not for a bilateral condition. Overall, 20 out of 42 studies reported the duration of hearing loss or deafness prior to implantation. Specifically, eight studies reported the duration of deafness, among which four reported the average (22.8, 3.2, 2.6, and 3.6) and three reported the range (0.3–41, 0.3–10, and 1–6) of years with deafness prior to implantation across subjects. Twelve studies reported duration of hearing loss, among which three studies reported the range in years of hearing loss across subjects (1–28, <10, <20) and nine studies reported the average in years of hearing loss across subjects (across studies: 22.2 ± 8.7).

**Hearing loss severity.** Pre-operative average pure-tone detection was reported in 16 out of 42 studies in various forms, either directly stating in the text the average PTA or supplying a pure-tone audiometric graphic from which we estimated the average PTA at three frequencies (0.5, 1, and 2 kHz). Note that depending on the graphic resolution, these values were not always accurate. Across these studies, we found that the average PTA was 89.5 dB (STD 12.5).

#### 3.4.2. Post-Operative Speech and Music Perception Outcomes

Different types of speech and music performance tests were used to determine post-operative outcomes. Thirty-three studies reported only speech outcomes, three studies performed both speech and music tests, and nine studies reported only music tests. The assessment tool(s) used in these publications pertaining only to music varied greatly. Six studies used surveys, questionnaires, and quality of life measures to assess performance. Four studies used music samples, music bursts, instrument identification, timbre, and intonation identification to assess outcomes. Two studies reported outcomes using validated music tests, specifically the Musical Sound Quality Impairments in Cochlear Implants (MUSHRA, Johns Hopkins University) and the Musical Sounds in Cochlear Implants perception test (MuSIC, Technical University Munich). Additional outcome assessment tools included objective measures and vocoded stimuli, i.e., synthesized signals that are thought to simulate, for a normal-hearing listener, the perception of speech as heard through a cochlear implant. In addition to a wide variety of outcome measures, we also found the aims of these studies were quite diverse, like the studies on speech perception outcomes ranged from assessing quality and enjoyment of sound to evaluating the impact of an online music training program. We found that most studies reported speech perception outcomes using the CNC word recognition test (*n* = 16), with AzBio, a sentence list test, mentioned in nine studies (see Figure 3). In addition, 94% of all speech performance tests were conducted under noise conditions.

Next, we specifically investigated the test conditions used within the studies that reported both CNC word recognition and AzBio sentence list tests. This was to assess whether results could be compared in a meta-analysis. We found that AzBio studies were performed adaptively and in each paper at a different presentation level (60 or 65 dB, with +5 or +10 dB SNR), which prohibited the possibility of summarizing the scores.

On the other hand, studies reporting CNC test results had more commonalities. We identified six studies that reported the exact same conditions in which the test was conducted: non-adaptive 60 dB SPL in quiet. From these studies, three studies were performed with CI systems by Cochlear and three studies with CI systems by MED-EL. In Table 1, we summarize the results reported for CNC in quiet in the respective studies. Note that some studies did not report the values directly in the text, and these needed to be extracted from the graphics; hence, some small inaccuracies should be expected.

To account for the different number of participants in each study, we performed weighted averaging of the scores by the number of participants (see Table 2). We synthesized the results for different intervals including 1 month, 3 months, 6 months, and 12 months following implant operation. Note that it was not possible to perform a meta-analysis of the effect sizes for these studies as some studies (Dillon, Buchmann, Buss, Canfarotta) did not report the necessary parameters (standard deviations—STD, standard errors of the mean—SEM). Notably, the results suggest that the differences between the CI systems in terms of speech outcomes in quiet are negligible. Again, differences may exist when noisy or adaptive conditions are tested, but data are not available to make such comparisons.

## 4. Discussion

In this scoping review, we analyzed the literature for recent studies (2015–2022) reporting speech and music performance outcomes in adults implanted with a CI system. Our aims were (1) to map the literature on speech and music performance outcomes and (2) to find whether studies have performed outcome comparisons between devices of different manufacturers. Our findings show that very few publications directly compared patient performance outcomes between manufacturers of CI systems. Similar findings have been recently reported [14] for a limited selection of medical devices. CI device comparisons across different manufacturers are rare not only within an implant center but also across centers.

The lack of performance-based manufacturer comparative publications is possibly due in part to the fact that there is no consensus among large CI programs regarding a systematic method of collecting outcome data. Differences in speech outcome measures (monosyllabic words, sentence in quiet, and sentence in noise), differences in presentation levels (adaptive, fixed dB, and various SNRs), and inclusion of other measures, such as quality of life (QoL) and music evaluation, vary significantly between CI centers. Such differences make data collation a challenging task. For instance, Carlson et al. [33] surveyed CI centers and found that 100% of responding clinics used AzBio sentences and 68% used speech-in-noise testing to determine candidacy. However, there was no consistency in the level of noise used. Some centers reported using a +10 SNR, others reported a +5 SNR, and a majority used some combination of the two. As with pre-operative assessment for candidacy, a lack of consistency is seen when measuring post-operative outcomes.

In addition, performance-based manufacturer comparisons demand large numbers of implanted patients to account for interindividual variability as well as other factors influencing performance outcomes (see above), which perhaps prohibit large CI centers from this task. However, evidence suggests that large CI centers in Germany and the USA perform speech perception tests as a routine procedure pre-implantation. For example, the FDA only approves clinical trials that use the CNC word recognition test as the primary measure for determining CI candidacy [34]. In addition, they recommend specific set-up for the test that could be potentially adopted by large CI centers in the USA. In Germany, the regulations are also specific. The Freiburger Test is recommended by the German society for Otolaryngology in the publication “Weissbuch: Cochlea-Implantat (CI)—Versorgung in Deutschland [35] (Überarbeitete 2. Auflage, 2021)”. In accordance with this recommendation, a large CI center in Kiel, Germany, conducted retrospective analyses of speech performance outcomes using the Freiburger Test [36] in 626 persons implanted with a CI in the years 2010–2015. While most patients were implanted with a CI device manufactured by Cochlear (*n* = 165), some patients were also implanted with devices by other manufacturers (MED-EL: *n* = 23, AB: *n* = 11). Despite the relatively low number of implants for comparisons between manufacturers, it is clear that data and methods are available for performing such meaningful comparisons, at least in Germany and the USA. Unfortunately, other countries, such as France, Spain, Netherlands, or the UK, have less specific regulations regarding CI candidacy, which subsequently leads to a strong variety in speech and music performance tests used [37].

Another example that data and methods are available to perform comparisons is a study conducted in Wurzburg, Germany [38]. In a retrospective data analysis involving 55 subjects, the Freiburger Numbers, Freiburger Monosyllable, and HSM sentences were examined at one, three, six, and twelve months, as well as at yearly follow-up appointments. Similar to the Kiel study mentioned above, the number of implants was too low to compare between manufacturers as they had only six subjects with either a Cochlear or AB device. Nonetheless, the presence of such data demonstrates that comparisons between device manufacturers are possible.

In the 42 publications that passed our selection criteria, we found that the vast majority mentioned a specific CI system manufacturer and 88% of those studies also mentioned the specific device used. In addition, we found six publications with >70 subjects, and one study included a sample of 150 subjects. With such large cohorts, inter-individual variability as well as other factors influencing performance may play a less significant role when averaging speech performance outcomes. Notably, sample sizes strongly varied between studies. We provided a power analysis for the comparison between CI systems of different manufacturers, which assessed the minimum number of subjects required to perform meaningful manufacturer comparisons to be 42, when considering between-group comparisons.

In terms of outcome assessment, we found that most studies used speech performance tests to assess the beneficial effect of CI systems. Several studies also used music performance tests to assess performance outcome following implantation. There was a strong variability in the tests used to quantify speech and music performance post-operatively. Nonetheless, we identified 16 studies that used the CNC test under different conditions. From these, six studies were conducted in the USA, which means that all subjects were English speakers, and the studies used similar CNC test conditions: non-adaptive 60 dB SPL in quiet. This allowed us to perform weighted averaging of CNC outcomes at different time intervals following implantation across the six studies. Note that summarizing the data across the studies using effect sizes was not possible since some of the studies did not report standard deviations or standard errors, which simply does not withstand good scientific practice. Notably, three studies featured MED-EL devices and three studies featured CI systems by Cochlear. The differences found were rather negligible and inconsistent; thus, no statements could be made regarding the superiority of any one specific device. Although CNC in quiet was most commonly used in the studies reported here, sentence tests such as AzBio are a better reflection of speech performance in real-life scenarios. However, as words in a sentence are associated with each other, it is impossible to detach the effect of context on hearing ability [39]. In addition, testing under a quiet condition is not valuable since a far more important outcome is comprehension of speech under noise conditions. Unfortunately, studies implementing sentence tests (examples) in noise varied significantly in terms of the specific test conditions, thus not allowing cross-study comparisons. Optimally, post-operative comparisons between devices should be performed within one CI center. As stated above, there is evidence that CI centers in Germany routinely perform the Freiburger test post-operatively at different time intervals. Such data could be leveraged to fill the knowledge gap in terms of reliable manufacturer comparisons. Note, however, that we found only three studies that used the Freiburg test, probably because most reports on the Freiburg test are from German CI centers and written in German and we excluded non-English journals.

Notably, in terms of music performance, we found extreme inconsistencies between studies: of the nine studies identified for in-depth review, no two studies used the same test to assess outcomes. Some of the parameters tested were often similar but not identical to those in standardized tests such as MuSIC [40,41,42]. As for speech perception tests, the music perception tests used varied in various features. This prohibited us from performing any kind of summary analysis on these outcomes. Notably, music tests to assess outcomes of CI implantation are relatively new and not anchored in any reimbursement regulation and, therefore, are less reported and less consistent. As music performance is a significant measure that strongly affects patients’ quality of life [5,43], we urge future studies to follow a validated music test.

It would also be important to investigate how cultural differences affect CI users’ ability to perceive music. Indeed, previous studies could link cultural aspects and different music perception parameters, such as pitch discrimination, melody, and rhythm [44,45]. These findings could assist with future collation of data across different CI centers around the world, as well as guide CI manufacturers in the adaptation of CI technology to achieve optimal music performance.

We tried to map the reasons regarding the feasibility of comparative studies between CI systems of different manufacturers. Firstly, it would be important to compare technologies of the same generations. Large implant centers have patients using multiple generation devices, which could make comparisons difficult. Secondly and as stated above, many subjects would be required to better control for inter-individual variability. Small-to-medium-size centers may not have enough subjects to draw a comparison. Thirdly, and specifically related to cross-center comparisons, the evaluation measures, presentation levels, signal-to-noise ratios, test intervals, and test language need to be consistent to make a valid comparison. In a retrospective study design, it is understandable that finding such consistency across centers is challenging. A prospective study would be easier to design but would also have its own challenges. Centers would need to implant the same generation technologies and match the subjects in terms of age, duration of deafness or hearing loss, and duration of implant use. Data collection would likely take several years to draw conclusive findings. Additionally, some patient-related parameters that would be important to consider when comparing outcomes were either not reported or varied between studies. Long durations of deafness and certain etiologies are examples that are associated with poorer outcomes and, therefore, should either be part of the exclusion criteria or matched pairs should be considered as they do not reflect the average population.

Although direct comparisons between manufacturers are scarce, we did find publications reporting performance outcomes in large numbers of subjects implanted with the same device. In these studies, data collection was very consistent and systematic. Patients were tested at defined test intervals using specific evaluation measures at the same presentation level and SNR. Potentially, large CI centers that have implanted a sufficient number of devices (see power analysis above) from different manufactures could conduct retrospective comparisons using available data. Alternatively, if center A with a large cohort reported outcomes with device X and center B, with an equally large cohort, reported outcomes with device Y, a comparison between device outcomes could be made. Probably the most valid comparison of speech and music performance outcome of different CI manufactures is in patients implanted with two different brands of CI [10]. As mentioned in the Introduction, most CI users implanted with two brands of CI have an apparent preference toward one device, which clearly shows the necessity of providing comparative outcome data to the great benefit of the CI community.

### Limitations of This Scoping Review

Some limitations of this scoping review need to be mentioned. First, we used specific search terms, “Cochlear implant outcomes adults” and “Cochlear implant music adults”, which may have impacted the extent of studies found. However, it should be noted that an initial search with different terms yielded similar results. The exclusion criteria for both patient indications and study types as well as publication language (English) might have also limited the number of publications available for review. Lastly, we restricted our search to recent publications from 2015 onward to be able to compare results of users with current generation technology. Reviewing literature published prior to 2015 would have produced more outcome measures for comparison but with outdated technology.

## 5. Conclusions

We found very few publications that compared speech and music outcomes across manufacturers. Performance data of different studies cannot be compared between manufacturers for various reasons, most notably being the variability in assessment measures and test conditions, as well as reporting bias. We argue that it is possible, however, to perform such quantitative comparisons. Data to do so should already exist, at least in large CI centers in Germany and the USA, but analyses have not been published despite the strong need by CI recipients and medical professionals. We therefore urge the community to make these data available. Future efforts should focus on forming a consensus regarding a systematic method for collecting outcome data following CI implantation, which will serve clinicians and prospective CI recipients with performance-based comparisons between different CI systems and lead to informed decision making. In addition, this information could drive innovation in device design as well as future developments of CI systems that are focused on patients’ experience. Lastly, systematically collected data could provide predictive information for clinicians regarding performance outcomes.

## Figures and Tables

**Table 1:** Overview of publications that collected CNC word scores at a presentation level of 60 dB SPL in quiet.

Paper	Manufacturer	Study Location	N-Subjects	1-Mo	3-Mo	6-Mo	12-Mo
Kelsall	Cochlear	Colorado, USA	100	-	56%	61%	65%
Dillon (FSP)	MED-EL	N. Carolina, USA	11	38%	50%	59%	-
Runge	Cochlear	Wisconsin, USA	38	-	49%	53%	57%
Buchman	Cochlear	Missouri, USA	96	-	-	61%	-
Buss	MED-EL	N. Carolina, USA	20	39%	43%	59%	57%
Canfarotta	MED-EL	N. Carolina, USA	19	49%	53%	62%	60%

**Table 2:** Weighted averaging of speech scores across manufacturers.

	1-Mo	3-Mo	6-Mo	12-Mo
MED-EL	42.6%	48.3%	60.1%	58.5%
Cochlear	-	54.1%	59.7%	62.8%

## Data Availability

The Excel spreadsheet can be accessed online at https://github.com/Syte-Institute/Publications/blob/6e8c92d0a70589e1ceefb34ba1134143d2cf1de9/230613_CI%20outcomes%20speech%20and%20music.xlsx (accessed on 27 September 2023).

## Supplementary Materials

The following supporting information can be downloaded at: https://www.mdpi.com/article/10.3390/audiolres13050067/s1, Table S1: Checklist; Table S2: CI outcomes speech and music.
